# Changes in deceleration capacity of heart rate and heart rate variability induced by ambient air pollution in individuals with coronary artery disease

**DOI:** 10.1186/1743-8977-7-29

**Published:** 2010-10-07

**Authors:** Alexandra Schneider, Regina Hampel, Angela Ibald-Mulli, Wojciech Zareba, Georg Schmidt, Raphael Schneider, Regina Rückerl, Jean Philippe Couderc, Betty Mykins, Günter Oberdörster, Gabriele Wölke, Mike Pitz, H -Erich Wichmann, Annette Peters

**Affiliations:** 1 Helmholtz Zentrum München, German Research Center for Environmental Health, Institute of Epidemiology, Neuherberg, Germany; 2 IBE Department of Epidemiology, Ludwig-Maximilians-University of Munich, Munich, Germany; 3 Cardiology Division, University of Rochester Medical Center, Rochester, NY, USA; 4 First Medical Clinic, Munich University of technology and German Heart Center Munich, Munich, Germany; 5 Medtronic Bakken Research Center, Maastricht, The Netherlands; 6 Department of Environmental Medicine, University of Rochester School of Medicine and Dentistry, Rochester, NY, USA; 7 University of Augsburg, Environmental Science Center, Augsburg, Germany; 8 Focus Network Nanoparticles and Health (NanoHealth), Helmholtz Zentrum München, German Research Center for Environmental Health, Neuherberg, Germany

## Abstract

**Background and Objective:**

Exposure to ambient particles has been shown to be responsible for cardiovascular effects, especially in elderly with cardiovascular disease. The study assessed the association between deceleration capacity (DC) as well as heart rate variability (HRV) and ambient particulate matter (PM) in patients with coronary artery disease (CAD).

**Methods:**

A prospective study with up to 12 repeated measurements was conducted in Erfurt, Germany, between October 2000 and April 2001 in 56 patients with physician-diagnosed ischemic heart disease, stable angina pectoris or prior myocardial infarction at an age of at least 50 years. Twenty-minute ECG recordings were obtained every two weeks and 24-hour ECG recordings every four weeks. Exposure to PM (size range from 10 nm to 2.5 μm), and elemental (EC) and organic (OC) carbon was measured. Additive mixed models were used to analyze the association between PM and ECG recordings.

**Results:**

The short-term recordings showed decrements in the high-frequency component of HRV as well as in RMSSD (root-mean-square of successive differences of NN intervals) in association with increments in EC and OC 0-23 hours prior to the recordings. The long-term recordings revealed decreased RMSSD and pNN50 (% of adjacent NN intervals that differed more than 50 ms) in association with EC and OC 24-47 hours prior to the recordings. In addition, highly significant effects were found for DC which decreased in association with PM_2.5_, EC and OC concurrent with the ECG recordings as well as with a lag of up to 47 hours.

**Conclusions:**

The analysis showed significant effects of ambient particulate air pollution on DC and HRV parameters reflecting parasympathetic modulation of the heart in patients with CAD. An air pollution-related decrease in parasympathetic tone as well as impaired heart rate deceleration capacity may contribute to an increased risk for cardiac morbidity and sudden cardiac death in vulnerable populations.

## Background

Epidemiological studies have shown an association between elevated levels of particulate air pollution and increased cardiovascular and respiratory morbidity and mortality among the elderly [1]. Furthermore, particulate air pollution has been found to be related to changes in autonomic nervous system function, the presence of cardiac arrhythmias as well as the onset of myocardial infarction (MI) [2-8].

Time- and frequency domain analysis of heart rate variability (HRV), which enables the non-invasive assessment of impaired autonomic nervous activity, can be used to identify patients at risk for cardiac death [9]. A decrease in HRV parameters is a strong risk factor for cardiac death in patients after MI and in those with chronic heart failure [10,11]. Furthermore, phase-rectified signal averaging (PRSA), developed by Bauer et al. (2006) [12], allows periodicities to be extracted from complex time-series that may include non-stationarities, noise and artifacts, as well as periodic components; while non-periodic components are eliminated. By applying PRSA on the heart signal, the proposed parameter "deceleration capacity" (DC) can be derived. DC describes the average behaviour of heart rate around phases when the heart slows down. According to the same authors, that impaired deceleration capacity is both a powerful and independent parameter for risk prediction in post-infarction individuals that is more accurate than conventional measures of heart rate variability [13]. Moreover, the authors believe that DC provides a measure of cardiac vagal modulations. Their findings show that the ability to slow the heart down is more clinically important than the ability to speed it up. To our knowledge, this marker has yet to be used in an epidemiological setting to analyze the adverse health effects of air pollution and may help to further disentangle the effects of vagal and sympathetic modulators on the heart.

In epidemiological studies, associations for HRV were seen with acute exposure to ambient PM_10 _(particle mass of particles < 10 μm) or PM_2.5 _(particle mass of particles < 2.5 μm). Most associations were rather immediate (within hours or on the same day of exposure) [5,14,15], but longer time-lags were also observed [3,16,17]. Moreover, some epidemiological studies have associated with ambient air particles in the ultrafine range (UFP, number count of particles < 1 μm) with cardiac morbidity and mortality [18-20] Due to their large surface area in relation to their volume, it has been speculated that UFP have a greater inflammatory effect than larger particles. In addition, it was observed that ultrafine particles could be translocated into extrapulmonary organs and systemic circulation [21-24], which led to the hypothesis that on a mass basis UFP might be more harmful than larger particles of the same chemical composition. Recently, a clinical study on the autonomic modulation of the heart was performed in which healthy volunteers were exposed to ultrafine carbon particles [25]. Though the changes in the ECG-derived parameters were small, observed trends indicated that some people might be particularly susceptible to UFP exposure.

The specific objective of this study was to evaluate the association between exposure to ambient fine and ultrafine ambient particles by analyzing repeatedly measured HRV parameters in individuals with coronary artery disease (CAD). To our knowledge, this study marked the first time that the rather new parameter DC, which goes beyond the commonly used HRV parameters, was included in an epidemiological setting to assess its association with ambient air pollution.

## Materials and methods

### Study population

The study was conducted in patients with CAD in Erfurt, Germany, between October 16^th ^2000 and April 27^th ^2001. The city, located 200 m above sea level and mainly surrounded by a 100 m high ridge except towards the North, had a population of approximately 200,000 at that time. These geographic conditions favor wintertime inversions, which result in elevated levels of ambient air pollution. Traffic, heating, energy production, and long-range transport are the major sources of ambient air pollution.

Study participants were recruited through a local cardiologist. They were required to be males aged 50 or more with physician-diagnosed ischemic heart disease, stable angina pectoris or prior MI (more than three months ago). Based on the study objectives, current smokers, individuals with pacemakers, bundle-branch block, type 1 diabetes, recent MI, bypass-surgery or balloon dilatation (less than three months ago) and patients on anti-coagulation therapy were excluded from participation. Of the 61 recruited subjects, 56 met the inclusion criteria for the ECG analyses. Three patients with bundle-branch blocks had to be excluded, as well as one who had constant arrhythmia and one who was not compliant. Written consent was obtained from each subject. The study protocol was approved by the German Ethics Commission "Bayerische Landesaerztekammer".

### Clinical visits

A total of 12 clinical visits were scheduled for each participant - one every two weeks on the same day at the same time of the day to control for weekly and diurnal patterns of the assessed parameters. Before the first examination a baseline questionnaire was administered to obtain demographic information, health status, pulmonary and cardiac symptoms, medication use, smoking history, exposure to environmental tobacco smoke (ETS), living conditions and indoor and outdoor sources of particulate air pollution. Each visit included a short interview to assess potential confounders and to collect information on current health status and changes in medication. Participants in addition kept a daily diary with information on times spent in smoke-filled rooms and times spent in traffic using a car, bus, tram or taxi.

### ECG recordings

ECGs were recorded with a 12-lead Mortara H12 recorder (Mortara Instrument, Milwaukee, USA) using a digital sampling rate of 180 samples/sec per channel. The short 20-minute ECG recordings included a 6-minute period of rest in supine position with spontaneous breathing. Every four weeks after the short ECG recordings had been made, the study subjects underwent 24-hour Holter monitoring and were advised to keep activity diaries, which included the time, duration and type of each activity during the monitoring period. Thus, during the six months of the protocol, each subject was supposed to have twelve short and six long Holter recordings paralleled by air pollution monitoring.

### ECG parameters

The ECG recordings were analyzed at the University of Rochester Medical Center, (Rochester, NY) for computing the HRV parameters and at the First Medical Clinic of the Munich University of Technology and German Heart Center Munich, Germany, for measuring DC. For the analysis, the first minute of the 6-minute period was discarded to avoid carry-over effects from previous minutes. For the 24-hour recordings, the entire recording was included in the analysis. Only normal sinus beats were used to calculate HRV - artifacts and ectopic beats were excluded after scanning and manual editing of the QRS complexes. Then, the tachograms (curves describing the length of the successive RR intervals across the analyzed period) were exported to be processed by computer algorithms providing both time and frequency domain HRV parameters. The list of HRV parameters included in this analysis was computed according to the current standard described in the recommendation of the Task Force of the European Society of Cardiology and the North American Society of Pacing and Electrophysiology [26].

Average heart rate (HR) is expressed in number of cardiac beats per minutes such as 60/RR when RR intervals are measured in seconds. The time domain HRV parameters included SDNN, RMSSD and pNN50 where SDNN is the standard deviation of RR intervals on a given period for all normal-to-normal beats (NN), RMSSD is the root means square of successive differences in RR values, and pNN50 is the percentage of adjacent RR intervals which differ more than 50 ms.

The frequency domain HRV parameters used in the analysis were computed using the power spectral density method of the tachograms based on fast Fourier transformation. The power of the estimated spectrum was measured into the so-called low-frequency (LF, 0.04-1.5 Hz) and high-frequency (HF, 0.15-0.40 Hz) bandwidths. These measurements were normalized (LFn and HFn) with LFn = LF/(TP-VLF) and HFn = HF/(TP-VLF) where VLF represents the energy in the very-low-frequency bands (0.003-0.04 Hz) and TP the power of the total spectrum. LFn and HFn have no unit (n.u.).

For the 5-minute ECGs we analyzed HR, HF, LF and RMSSD. In association with air pollution, only the normalized frequency domain parameters HFn and LFn were used. For the 24-hour ECGs HR, SDNN, RMSSD and pNN50 were computed.

To assess DC from the 24-hour ECGs, the signal processing technique of PRSA was used to process sequences of NN intervals from pre-discharge Holter recordings. The technique provided separate characterization of deceleration-related and acceleration-related modulations, quantified by deceleration capacity and acceleration capacity. Details on the methodology can be found in Bauer et al. (2006)[12]. For the analysis with air pollution, only DC was used as Bauer et al. (2006) [13] showed that mechanisms which slow the heart down are clinically more important than those that speed it up and that DC was suitable as a screening method with high prognostic value.

### Air pollution monitoring

The concentrations of ambient air pollutants were measured at a fixed monitoring site representing urban background levels. The Erfurt Aerosol Measurement Site and its equipment have been described in detail elsewhere [27]. Briefly, we sampled the particle size distribution with a mobile aerosol spectrometer for particles of a size range between 0.01 μm and 2.5 μm. Hourly number concentrations for ultrafine particles (UFP, 0.01-0.1 μm) were calculated from the spectra. Moreover, hourly mass concentrations of particles with a size range between 0.01 μm and 2.5 μm (PM_2.5_) were calculated assuming spherical particles with an estimated mean density of 1.53 g/cm^3^. Elemental carbon (EC) and organic carbon (OC) were determined hourly from an ambient carbon monitor (5400, R&P, Inc., Albany, NY, USA) only after December 17^th ^2000. The continuous data on meteorological variables such as temperature, barometric pressure and relative humidity were collected from existing networks [27]. Missing values for UFP and PM_2.5_, temperature and relative humidity were either replaced based on corrected parallel measurements (temperature, relative humidity) or imputed by semiparametric regression models based on data from other devices (UFP, PM_2.5_) [27]. The semiparametric model allowed for the inclusion of a smooth function of time to account for temporal variations in the measurements. The Goodness of Fit values were 0.94 and 0.88 for the UFP regression model and for the PM_2.5 _imputations, respectively. Missing values for OC and EC were not imputed. To determine exposure to air pollutants prior to the recording of the short-term ECG individual 0-5, 6-11, 12-17, 18-23, 0-23 (lag 0), 24-47 (lag 1), 48-71 (lag 2), 72-95 (lag 3), 96-119 (lag 4) and 0-119 (5-day average) hour averages of air pollution parameters were calculated based on the starting time of the recordings. For the 24-hour Holter recordings the time at the end of the recording was taken to determine exposure to air pollutants concurrent and prior to the ECG-recording (lag 0 to lag 4). Average concentrations of the air pollutants were calculated when at least 2/3 of the hourly measurements were available.

### Statistical analysis

Data were analyzed using the statistical package SAS Version 9.1 (SAS Institute Inc., Cary, NC, USA). A descriptive analysis of the characteristics of the study participants was based on data obtained through the baseline questionnaire. Mixed models with a random participant effect and covariance structure "compound symmetry" were used to analyze the association between air pollutants and ECG parameters taking into account the repeated measurements over time for each individual. Since measurements took place two weeks apart no adjustments for autocorrelation were necessary. RMSSD of the short - as well as of the long-term recordings was log-transformed since the residuals were otherwise not normally distributed. Non-parametric smooth functions (penalized splines) were used to explore the shape of the association between confounders such as trend or meteorological variables and the dependent variable. In addition, day of the week was also considered as a potential confounder. Model fit was based on minimizing the Akaike Information Criterion. Models were built for each ECG parameter separately. The selected confounder model for each ECG parameter can be found in the Additional File 1.

As a sensitivity analysis we re-analyzed the effects for PM_2.5 _and UFP using the same reduced time-period for which EC and OC were available. Moreover, we did a further sensitivity analysis by excluding participants who were taking anti-arrhythmic medication.

For effect modification, we considered body mass index (BMI < 30 kg/m^2 ^vs. ≥30 kg/m^2^), smoking history (ex-smokers vs. never-smokers) and intake of beta-adrenergic receptor blockers (yes vs. no). Information from the daily diaries about times spent in smoke-filled rooms and times spent in traffic for the times during the ECG as well as the lagged times was coded binary (at least one hour vs. less than one hour) and used for interaction analysis of the long-term ECGs.

Effect estimates are presented as percent change from the mean (geometric mean for RMSSD, arithmetic mean for all other ECG-variables) together with 95%-confidence intervals based on an interquartile range (IQR: difference between the first and the third quartile) increase in air pollution concentration.

## Results

### Study population

Descriptive characteristics of the study population are given in Table 1. The study group consisted of 56 male volunteers with an age range of 52 to 76 years and a BMI range of 22 to 38 kg/m^2^. The majority of the participants had prior MI and all of them had stable ischemic heart disease. All individuals were current non-smokers, but almost three quarters had a history of smoking. More than two-thirds of the participants stated that they were hypertensive. They were treated mainly with beta-adrenergic receptor blockers or angiotensin-converting enzyme (ACE)-inhibitors, only two patients were on anti-arrhythmic therapy. Over three quarters of the participants took acetylsalicylic acid (aspirin). Medication was maintained without changes during the study except for 10 patients (18%) who had an update in medication (either removed or added prescription).

**Table 1:** Description of the study population: 56 male individuals with a history of coronary artery disease.

Clinical Characteristics		Mean (± SD)
Age [years]		66 (± 6)
Body mass index (BMI) [kg/m^2^]		28 (± 4)
		**Total number N (%)**
History of myocardial infarction		43 (77)
Revascularization (CABG/PTCA^a^)		48 (86)
Type 2 diabetes mellitus		12 (21)
Hypertension^b^		39 (70)
NYHA≥ II		17 (30)
COPD		5 (9)
Occupational status:	- Work part time or full time	4 (7)
	- Retired	49 (88)
	- Unable to work or unemployed	3 (5)
	- Exposed to toxic gases, dust or fumes at work	1 (2)
Smoking:	- Never-smoker	15 (27)
	- Ex-smoker	41 (73)
Medication use:	- Beta-adrenergic receptor blockers	43 (77)
	- Anti-arrhythmic therapy	2 (4)
	- ACE-inhibitors	30 (54)
	- Calcium-blockers	17 (30)
	- Nitrates	24 (43)
	- Acetylsalicylic acid (aspirin)	46 (82)
	- Statins and Fibrates	29 (52)

SD: standard deviation; CABG: coronary artery bypass graft surgery; PTCA: percutaneous transluminal coronary angioplasty; NYHA: New York Health Association functional classification; COPD: chronic obstructive pulmonary disease; ACE: angiotensin-converting enzyme.

^a^Both procedures had to be performed more than three months prior to enrolment.

^b^Information from self-administered baseline questionnaire.

### ECG parameters

Of the targeted 672 clinical visits with 336 planned long-term ECG recordings, 625 5-minute ECG recordings and 254 24-hour recordings were available for the analysis of HRV after removing recordings based on exclusion criteria or poor quality. Table 2 shows the descriptive analysis of the ECG parameters across all observations for both the short-term as well as the long-term recordings. For the 5-minute ECGs the correlation between the markers was low to moderate with the highest value being 0.7 between HF power and LF power. On average the 24-hour ECGs were slightly higher correlated; the highest correlation was found between RMSSD and pNN50 with 0.8. As expected, the deceleration capacity was negatively correlated with HR (r = -0.7).

**Table 2:** Description and correlation of analyzed ECG-Parameters.

**Variable**	**N**	**Mean (± SD)**	**Min**.	**25%**	**Median**	**75%**	**Max**.	**IQR**	**Spearman correlation coefficient**^**a**^				
	
**5 minutes spontaneous breathing in supine position**													
									**HR**	**HF power**	**HFn**	**LF power**	**LFn**
									
HR [beats/min]	625	64.7 (± 9.9)	46.6	56.9	63.1	70.1	103.5	13.2	1				
HF power [ms^2^]	625	669.9 (± 3348.4)	3.6	16.4	40.4	202.4	70263	185.9	-0.4	1			
HFn [n.u.]	625	0.2 (± 0.2)	0.0	0.1	0.2	0.3	0.9	0.3	-0.1	0.5	1		
LF power [ms^2^]	625	519.2 (± 1378.3)	3.3	63.4	145.5	357.8	21807.3	294.4	-0.4	0.7	0.1	1	
LFn [n.u.]	625	0.4 (± 0.2)	0.0	0.2	0.4	0.6	1.0	0.3	-0.2	-0.1	0.30	0.3	1
RMSSD [ms]	625	40.8 (± 52.9)	4.5	14.7	22.3	40.7	462.4	26.0	-0.4	0.7	0.4	0.6	0.0
													
**24-hour recordings**													
									**HR**	**SDNN**	**RMSSD**	**pNN50**	
									
HR [beats/min]	254	69.3 (± 9.0)	50.4	62.7	69.0	74.7	94.2	12.0	1				
SDNN [ms]	254	124.9 (± 33.6)	59.8	101.3	122.6	147.0	222.7	45.7	-0.4	1			
RMSSD [ms]	254	42.9 (± 34.9)	9.9	22.2	31.3	49.8	292.1	27.6	-0.5	0.4	1		
pNN50 [%]	254	2.8 (± 3.9)	0.0	0.0	1.0	4.0	18.0	4.0	-0.5	0.6	0.8	1	
DC [ms]	254	5.6 (± 2.6)	0.3	3.8	5.3	7	21.1	3.2	-0.7	0.4	0.4	0.3	

SD: standard deviation; IQR: interquartile range; HR: heart rate, HF: high-frequency, HFn: normalized high-frequency; n.u.: no unit; LF: low-frequency, LFn: normalized low-frequency; RMSSD: square root of the mean square of successive differences of normal-to-normal (NN) intervals, SDNN: standard deviation of NN intervals, pNN50: percentage of adjacent NN intervals which differ more than 50 ms, DC: deceleration capacity.

^a^Median of patient-specific Spearman correlation coefficients.

### Air pollutants

A description of particulate and gaseous air pollutants as well as of meteorological variables is shown in Table 3. The time-series of the study period for UFP, PM_2.5 _and temperature can be found in Henneberger et al. (2005) [28]. Mean 24-hour concentrations of PM_2.5 _were 20 μg/m^3^, which is above annual average U.S. National Ambient Air Quality Standard (NAAQS) of 15 μg/m^3^, but below the new suggested EU Air Quality Standards directive of 25 μg/m^3 ^to be met by January 2015. On 29 days of the study period (15%, all in the winter) 24-hour PM_2.5 _concentrations were above the current 24-hour U.S. NAAQS of 35 μg/m^3 ^for PM_2.5_. So far, no EU directive exists regarding 24-hour averages.

**Table 3:** Daily concentration of air pollutants and meteorological variables between October 12^th ^2000 and April 27^th ^2001.

Variable	N	Mean (± SD)	**Min**.	25%	Median	75%	**Max**.	IQR	Spearman correlation coefficient					
									**PM**_**2.5**_	**UFP**	**EC**	**OC**	**Temperature**	**Barometric pressure**
	
PM_2.5 _[μg/m^3^]^a^	198	20.3 (± 14.8)	2.8	10.0	15.3	26.2	84.0	16.2	1					
UFP [n/cm^3^]^b^	198	11639 (± 5849)	2485	6967	10717	15299	28369	8331	0.5	1				
EC [μg/m^3^]^c^	113	2.3 (± 2.1)	0.2	0.9	1.6	2.7	10.4	1.8	0.8	0.7	1			
OC [μg/m^3^]^c^	113	1.4 (± 0.6)	0.3	1.0	1.3	1.7	3.4	0.7	0.7	0.6	0.9	1		
Temperature [°C]^d^	198	4.1 (± 4.8)	-10.8	-0.6	4.4	6.6	13.2	7.1	-0.5	-0.2	-0.4	-0.3	1	
Barometric pressure [hPa]	198	973.2 (± 9.8)	949.8	966.2	972.9	980.0	995.6	13.7	0.3	0.1	0.2	0.1	-0.1	1
Relative humidity [%]^d^	198	83.5 (± 8.9)	56.5	78.5	84.5	89.1	100.0	10.6	0.5	0.2	0.4	0.3	-0.4	0.1

SD: standard deviation; IQR: interquartile range; PM_2.5_: mass concentration of particles < 2.5 μm diameter; UFP: number concentration of particles between 0.01 and 0.1 μm diameter; EC, OC: elemental and organic carbon.

^a^715 (15%) of the hourly measurements were imputed.

^b^621 (13%) of the hourly measurements were imputed.

^c^Measurements started December 18^th ^2000.

^d^24 (0.5%) of the hourly measurements were imputed.

### Association between air pollution and ECG parameters

**Figure 1:**
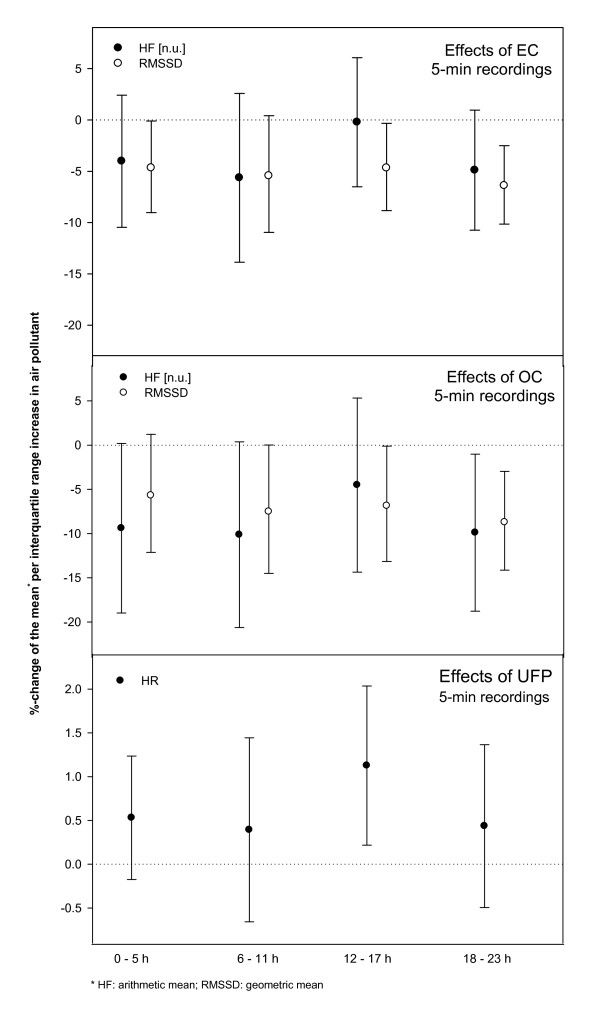
**Effect estimates (with 95%-confidence intervals) of particulate air pollution for 6-hour moving averages on heart rate and heart rate variability parameters of the 5-minute recordings per interquartile range increase in the respective pollutant**.

Regression of frequency domain HRV parameters and RMSSD during the 5-minute period of spontaneous breathing in supine position revealed decrements in RMSSD and HFn within the first 24 hours of exposure associated with increments in EC and OC (Table 4). These decrements were found to be quite consistent over the four 6-hour lag-periods (Figure 1). For HR a slight increase was seen only in association with UFP in the 12-17 hours prior to the recording (Figure 1). No effects were observed for LFn or in association with PM_2.5_.

**Table 4:** Effect estimates (with 95%-confidence intervals) of particulate air pollution on heart rate variability parameters per interquartile range increase in the respective pollutant.

		**PM**_**2.5**_	UFP	EC	OC
	
	Hours before recording				
**5 minutes of spontaneous breathing in supine position**	**HFn**	%-change of the arithmetic mean per increase in IQR pollutant (95%-CI)			
	0 - 23 h	-3.6 (-11.6;4.2)	-2.2 (-10.8;6.3)	-5.1 (-12.6;2.4)	-9.9* (-19.2;-0.6)
	24 - 47 h	0.0 (-7.0;7.1)	2.1 (-7.1;6.3)	-1.0 (-8.3;6.3)	-2.7 (-11.7;6.4)
					
	**RMSSD**	%-change of the geometric mean per increase in IQR pollutant (95%-CI)			
	0 - 23 h	-3.5 (-8.9;2.2)	-0.4 (-6.5;6.0)	-7.2** (-12.0;-2.1)	-8.0* (-14.0;-1.7)
	24 - 47 h	-0.7 (-5.8;4.7)	0.9 (-5.8;8.0)	-2.9 (-7.8;2.3)	-2.5 (-8.5;4.0)

					
**24-hour recordings**	**RMSSD**	%-change of the geometric mean per increase in IQR pollutant (95%-CI)			
	concurrent	-2.5 (-8.9;4.3)	1.9 (-4.1;8.4)	-2.9 (-8.0;2.4)	-1.7 (-8.2;5.3)
	0 - 23 h	-3.7 (-10.6;3.8)	2.3 (-4.3;9.3)	-2.7 (-8.4;3.3)	-1.3 (-8.1;6.1)
	24 - 47 h	-6.0 (-12.6;1.1)	-2.1 (-8.8;5.2)	-5.5* (-10.7;-0.1)	-5.8 (-12.1;0.9)
					
	**pNN50**	%-change of the arithmetic mean per increase in IQR pollutant (95%-CI)			
	concurrent	0.6 (-14.9;16.1)	5.5 (-8.4;19.3)	-4.7 (-17.1;7.6)	-5.9 (-22.0;10.2)
	0 - 23 h	-6.4 (-23.1;10.2)	1.6 (-13.5;16.7)	-9.6 (-23.0;3.9)	-14.5 (-30.3;1.2)
	24 - 47 h	-16.0 (-32.4;0.4)	-7.5 (-23.8;8.8)	-13.8* (26.6;-1.1)	-17.5* (-33.0;-2.1)
					
	**DC**	%-change of the arithmetic mean per increase in IQR pollutant (95%-CI)			
	concurrent	-6.4 (-13.1;0.3)	-0.8 (-7.1;5.4)	-9.1** (-15.4;-2.8)	-11.5** (-19.4;-3.6)
	0 - 23 h	-9.9* (-17.4;-2.3)	-3.0 (-9.6;3.6)	-11.5** (-18.1;-4.9)	-14.2** (-22.2;-6.2)
	24 - 47 h	-11.4** (-19.4;-3.7)	-5.6 (-13.2;2.0)	-10.4** (-17.3;-3.5)	-11.4** (-19.4;-3.4)

PM_2.5_: mass concentration of particles < 2.5 μm diameter; UFP: number concentration of particles between 0.01 and 0.1 μm diameter; EC, OC: elemental and organic carbon; HFn: normalized high-frequency; IQR: interquartile range; CI: confidence interval; RMSSD: square root of the mean square of successive differences of normal-to-normal (NN) intervals, pNN50: percentage of adjacent NN intervals which differ more than 50 ms, DC: deceleration capacity.

* p < 0.05 (significant), ** p < 0.01 (highly significant).

**Figure 2:**
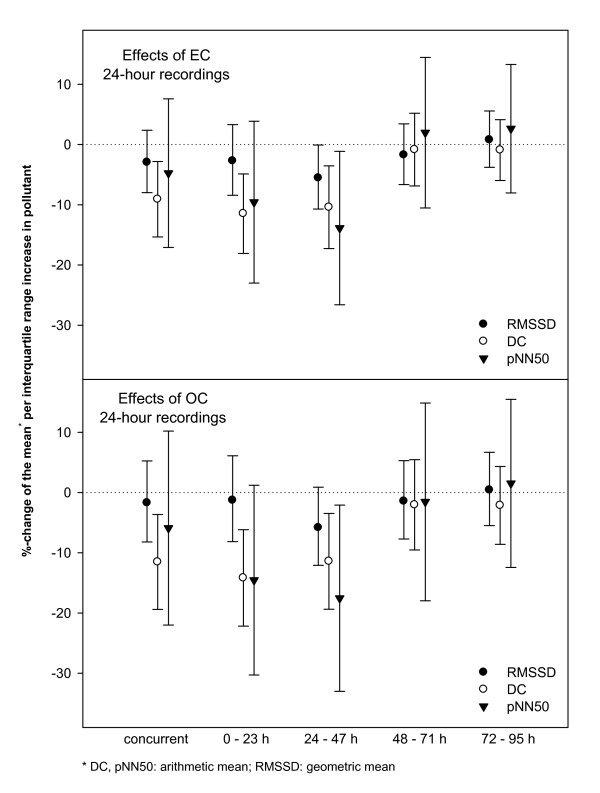
**Effect estimates (with 95%-confidence intervals) of OC and EC for 24-hour moving averages on deceleration capacity and heart rate variability parameters of the 24-hour recordings per interquartile range increase in the respective pollutant**.

Results of the 24-hour ECG recordings in association with 24-hour mean particulate air pollutants are given in Table 4. RMSSD decreased in association with increasing EC and OC averages during the 24-47 hours before the ECG recording. In the same time-frame pNN50 showed even stronger decreases - also with EC and OC. In addition, DC decreased consistently in association with concurrent PM_2.5_, EC and OC averages as well as with lag 0 and lag 1 (Figure 2). DC-effects were highly significant and most consistent over different time-lags. No effects were found for HR or SDNN or in association with UFP.

The sensitivity analyses for the effects of PM_2.5 _and UFP in the same reduced time-period in which EC and OC were available showed no appreciable changes in effect estimates. On average, the associations became even slightly stronger but with wider confidence intervals due to less observations in the analyses. For DC, the same effects as in the main analyses remained significant. The sensitivity analyses excluding the two participants on anti-arrhythmic therapy (23 observations for the 5-minute ECGs and 7 observations for the 24-hour ECGs) showed slightly smaller effect estimates with some effects changing from significant to borderline significant. However, the order of magnitude remained the same for all effect estimates and especially all of the DC-effects remained very robust.

### Effect modification analysis

No difference could be found between obese and non-obese individuals with regard to air pollution health effects. Also smoking history and beta-adrenergic receptor blocker intake seemed not to substantially alter the observed associations. For the 24-hour recordings, the pNN50-effect seen with the EC- and OC-averages of 24-47 hours before the recording showed a trend towards a stronger effect in individuals not taking beta-adrenergic receptor blockers (data not shown). The diary data showed that during the 254 conducted long-term ECG measurements 22 participants had stayed in smoke-filled rooms for at least one hour whereas 215 participants had not been exposed to ETS and 17 observations had missing information. When using this data on ETS exposures, no significant interaction with air pollution was found. With regard to times spent in traffic, 68 participants spent less than one hour in traffic during their long-term ECG, 169 spent more than one hour in traffic and 17 observations had missing information. Participants spending more than one hour in traffic showed a trend towards stronger effects estimates especially in DC and pNN50 (Additional File 2).

## Discussion

Our analyses showed an immediate decrease in HFn and RMSSD in the 5-minute ECG-recordings for a period of spontaneous breathing in supine position, associated with increased levels of ambient air particles measured as EC and OC. This indicated a shift in the sympatho-vagal balance towards vagal withdrawal. Furthermore, in the 24-hour recordings, we observed a delayed decrease in RMSSD as well as in pNN50 for the same pollutants. In addition, deceleration capacity was found to decrease highly significantly at the same time as the 24-hour PM_2.5_, OC and EC averages increased and stayed decreased for up to 48 hours. Overall, our results in patients with CAD indicate a consistent decrease in parameters of HRV within 48 hours of exposure to ambient particles for all participants regardless of their obesity status, their history of smoking or their beta-adrenergic receptor blocker intake. Our findings suggest that inhaled particles may alter the balance between the sympathetic and parasympathetic control of the heart, resulting in a stress response that potentially leads to arrhythmias [29].

### Air pollution and HRV

Animal data support the hypothesis of the alteration of the autonomic nervous system by air pollution [30-32]. Evidence from epidemiological studies on the effects of ambient air pollution on HRV suggest an increase in HR [33-35] and a decrease in SDNN and RMSSD as well as in HF associated with particulate air pollution in healthy elderly individuals [5,15,16,33,35-39]. Effects were seen with either ambient or personal exposure to PM_2.5_, mainly within hours or on the same day of exposure. Clinical exposure studies also support these findings: Devlin et al. (2003) [40] reported that elderly subjects experienced significant decreases in HRV immediately following exposure to concentrated air pollution particles - persisting at least 24 hours after exposure for some HRV parameters. However, positive associations between air pollutants and HRV parameters have also been shown [41,42]. However, whilst there are some inconsistencies between studies, the general consensus is that HRV is impaired by exposure to air pollutants.

#### Susceptible subgroups

Although it has been hypothesized that individuals with cardiovascular disease may be more susceptible to ambient air pollution, no sound body of evidence exists. Stronger associations of particulate matter and HRV in subjects with ischemic heart disease and hypertension have been shown by Park et al. (2005) [16]. Inconsistent results were found in a European multi-centre study in CAD patients: an increase in HF and reduced LF/HF in Erfurt, Germany, and a decreased HF and an increased LF/HF in Helsinki, Finland [43]. Wheeler et al. (2006) [17] found a negative relationship between SDNN and PM_2.5 _in patients with cardiovascular disease whereas patients with pulmonary disease exhibited an inverse association. Furthermore, in a panel of coronary heart disease patients de Hartog et al. (2009) [3] observed associations between HRV and outdoor PM_2.5 _only in the subgroup of patients without intake of beta-adrenergic receptor blockers at 2- and 3-day lags. Beta-blockers can on one hand be seen as medication that stabilizes heart rate and reduces blood pressure. They are associated with bradi-cardiac effects and increased parasympathetic innervation. But on the other hand, the medication intake could be interpreted as a sign for the degree of sickness of people. And sicker people might be more susceptible to air pollution. We speculate that this could be the reason why we do not find strong influences v of beta-adrenergic receptor blockers in our analyses. Furthermore, differences in timing as well as different directions of the HRV changes might have been due to the heterogeneity of the study populations or to the particle sizes and composition in the various cited studies.

#### Particle components

Chuang et al. (2005) [44] who analyzed the association between particulate matter of different particle sizes and HRV in cardiac and hypertensive patients found that only PM in the size range from 0.3 to 1.0 μm exhibited significant effects on HRV parameters. In our study, we mainly found associations between EC or OC and changes in HRV. Atmospheric carbonaceous particles are an important component of PM_2.5 _and their main fractions are EC and OC. EC is emitted directly from combustion sources and can be seen as an effective indicator of primary anthropogenic air pollutants. OC, on the other hand, can not only be emitted from primary emission sources (primary origin can e.g. be benzene, toluene, ethylbenzene, xylene or polycyclic aromatic hydrocarbons), it can also be produced from volatile organic compound gas-to-particle conversion processes occurring in the atmosphere. Particulate emissions from diesel engines are composed predominantly of EC and OC. Diesel exhaust particles contain a solid carbon core that can aggregate redox active organics and inorganics and can lead to the generation of reactive oxygen species. It has been shown in various epidemiological studies so far that these soot/black smoke particles have a detrimental effect on human health [45]. A previous analysis in the same study population [28] demonstrated repolarization changes associated with EC and OC.

Furthermore, a study on heart rate variability among elderly by Schwartz et al. (2005) found stronger effects with traffic-related particles (black carbon) [46]. In the present study we also observed a tendency towards stronger effects in particular with DC and pNN50 when participants had marked in their diaries that they had spent more than one hour in traffic. This provides an indication that personal activities may modify cumulative personal exposure or its composition leading to more or less detrimental effects.

### Mechanistic background of air pollution effects

Several mechanisms explaining associations between ambient air pollution and cardiovascular effects have been suggested. Ambient air particles might activate neural mechanisms affecting the autonomic regulation of the heart responding through direct reflexes from irritant receptors in the airways [47]. Other potential mechanisms leading to a shift in sympatho-vagal balance could be a local or systemic inflammatory response triggering an acute phase reaction, a change in systemic vascular tone (endothelial dysfunction) or an ischemic response in the myocardium as suggested by Utell et al. (2002) [48]. Furthermore, altered ion-channel functions in myocardial cells could lead to cardiac malfunction triggered by air pollution [49]. According to Graff et al. (2004) [50] relative metal composition largely determines the cardiac effects of particles as metals alter the expression of ion channels and sarcolemmal proteins relevant to electrical remodeling. Other epidemiological studies suggest an acute phase reaction leading to adverse cardiac outcomes. Several authors worldwide have shown increases in plasma viscosity and inflammatory and coagulation markers in association with air pollution [51-55] - these were also shown for this study population [56].

Particles are also known to induce oxidative stress [57]. Inhaled particles may on one hand lead to local oxygen radical production in the lung due to the organic components on the particles or by transition metal catalyzed Fenton-reactions; on the other hand, ultrafine particles can be translocated into the blood circulation and produce oxygen radicals [21-24] due to their surface chemistry related properties through redox-sensitive pathways [57]. A study by Schwartz et al. (2005) [58] concluded that the effects of PM_2.5 _on HF seemed to be mediated by reactive oxygen species which increased oxidative stress. Oxidative stress can initiate further cellular responses, thereby contributing to the pathogenesis of PM-induced disease [59].

To complete the picture of air pollution-related cardiovascular effects that might result in a shift of sympatho-vagal balance, evidence also exists that higher ambient air particle concentrations are associated with an ischemic response [19,60] and with endothelial dysfunction [61,62].

### Implications of impaired HRV and DC

Autonomic imbalance is a major contributor to the triggering of cardiac arrhythmias and, as a consequence, to the incidence of sudden cardiac death [63]. Primarily reduced, but also increased HRV has long been recognized as a marker for cardiac mortality in high risk or elderly populations [26,64-67]. Additionally, it has been shown that HRV from 24-hour recordings was a stronger predictor of death due to chronic heart failure than other conventional clinical measurements [11]. Moreover, Bauer and colleagues (2006) [13] were able to show that the relatively new parameter DC is a powerful predictor of mortality after MI and is more accurate in its predictive ability than conventional measures of HRV. Patients with preserved acceleration capacity but a lack of deceleration capacity have been found to have a poorer outlook with regard to cardiovascular mortality.

Our study showed a decrease in HF, RMSSD and DC, all reflecting reduced vagal tone. DC characterizes the average capacity of the heart to decelerate the cardiac rhythm from one beat to the next and therefore visualizes the oscillations of heart rate with regard to the ability to slow the heart down. It indicates the overall deceleration capacity of the sinus rhythm, without necessarily being linked to one particular physiological regulation process (e.g. respiratory, baroreflex mediated or circadian). In addition, it is speculated that distinguishing between deceleration and acceleration capacity may be more successful in separating vagal and sympathetic modulators than other HRV parameters. Clinical and experimental studies indicate a cardioprotective role of vagal activity [68-70] and that vagal withdrawal can produce myocardial ischemia and possibly also be involved in the genesis of ischemic events [71,72].

### Strengths and limitations

The longitudinal approach with multiple observations per subject had the advantage of an almost complete follow up in all 56 participants over a period of six months with each subject acting as his own control. Since ECG parameters differ from person to person a random effect for each participant was included in the regression model, thus allowing adjustments for interpersonal differences and medication use of the participants. Although patients with CAD are likely to be more susceptible to air pollution, the association may be blunted by medication intake whereby medication may not only be a confounder but also an intermediate variable, and thus difficult to control for. A further strength was the analysis of two ECG-regimes, the 5-minute resting ECG as well as the 24-hour ECG. Both measurements represented very different information as for example in the 24-hour ECG the participants could have been physically active or in traffic concurrent to the ECG measurement. Moreover, the nocturnal period was included in the 24-hour ECG during which people in general have a very different autonomic regulation and which is mainly driven by the parasympathetic component.

Misclassification of air pollution exposure is another potential source of bias especially in time-series studies [73]. Factors such as wind direction, climatic conditions, long-range transport and distances from sources affect personal exposure patterns to pollutants from ambient sources. But any exposure misclassification would be expected to be non-differential and thus to bias the estimates towards the null. Although UFP may be more reactive than the larger particles, no significant associations were observed. UFP are usually freshly generated particles and agglomerate rather fast. Therefore the measurement of individual exposure to UFP by a central monitor is prone to misclassification which can bias the results towards zero. This would partly explain the lack of effects associated with particles in the ultrafine range. In addition, the times spent in traffic unfortunately did only account for using a car, bus, tram or taxi, but not for times as a pedestrian or on a bicycle. Therefore, possibly even more observations would have counted as spent partly in traffic which may have resulted in clearer differences between the two subgroups in our interaction analysis.

## Conclusions

This is one of the few epidemiological studies to report associations of particulate air pollution on HRV in patients with CAD, and to our knowledge, the first to include the relatively new parameter DC. A decrease in HF and RMSSD in the short-term recordings and a decrease in RMSSD, pNN50 and DC in the long-term recordings were associated with elevated concentrations of EC and OC, both mainly indicators of vehicle traffic-related air pollution. Exposures immediately and up to 48 hours before the recordings were responsible for these effects. The clinical relevance of the effects of ambient air particles on these autonomic function parameters is not yet established and the exact pathomechanisms responsible for these associations need to be elucidated. An alteration in such parameters in association with acute exposure to ambient air particles may be an indicator for impaired autonomic function, potentially leading in turn to adverse cardiac events in vulnerable people as indicated by the results of epidemiological studies that have shown increases in cardiac mortality and morbidity associated with decreased HRV and DC in people with heart disease.

## List of abbreviations

CAD: coronary artery disease; DC: deceleration capacity; EC: elemental carbon; ECG: electrocardiogram; ETS: environmental tobacco smoke; HF: high-frequency power (0.15 - 0.40 Hz); HFN: normalized high-frequency; HR: heart rate; HRV: heart rate variability; LF: low-frequency power (0.04 - 1.5 Hz); LFN: normalized low-frequency; MI: myocardial infarction; NN: normal-to-normal beats; N.U.: no unit; OC: organic carbon; PM_2.5_: particulate matter less than 2.5 μm in diameter; PNN50: percentage of adjacent NN intervals which differ more than 50 ms; PRSA: phase-rectified signal averaging; SDNN: standard deviation of NN intervals; RMSSD: square root of the mean square of successive differences of NN intervals; UFP: ultrafine particles, particles less than 0.1 μm in diameter;

## Competing interests

The authors declare that they have no competing interests.

## Authors' contributions

AS was involved in the analysis of the study and in the interpretation of the results. She also drafted the manuscript; RH performed the statistical analyses, created tables and figures for the manuscript and reviewed the manuscript critically; AIM was involved in the planning, conduction and analysis of the study. In addition, she provided the basis for the manuscript with her dissertation thesis and revised the manuscript critically; WZ was substantially involved in the analysis of the ECG data (except for the DC parameter) and reviewed the manuscript critically; GS was involved in the data acquisition and analysis of the DC parameter and reviewed the manuscript critically; RS performed the analysis of the ECG data with regard to the DC parameter and reviewed the manuscript critically; RR worked on the acquisition of the data, was involved in the interpretation of the data and revised the manuscript critically; JPC was substantially involved in the analysis of the ECG data (except for the DC parameter) and reviewed the manuscript critically; BM performed the ECG analysis (except for the DC parameter) and revised the manuscript critically; GO made substantial contribution to the design of the study and reviewed the manuscript critically; GW was substantially involved in the data acquisition during the field phase and was responsible for quality control and quality assurance. She reviewed the manuscript critically; MP was involved in the acquisition of the air pollution data and reviewed the manuscript critically; HEW was substantially involved in the design of the study and reviewed the manuscript critically; AP was substantially involved in the design of the study, the data acquisition and the interpretation of the results and reviewed the manuscript critically;

All authors read and approved the final manuscript.

## Supplementary Material

Additional file 1 **Selected confounder models for the ECG-parameters in the short-term and the long-term recordings**. Click here for file

Additional file 2 **Effect estimates with 95%-confidence intervals based on interquartile range increases in air pollutants for the interaction analyses with times spent in traffic (using a car, bus, tram or taxi)**. Click here for file

## References

[B1] Pope CA III Dockery DW Health effects of fine particulate air pollution: lines that connect J Air Waste Manag Assoc 2006 56 6 709 742 16805397 10.1080/10473289.2006.10464485

[B2] Berger A Zareba W Schneider A Ruckerl R Ibald-Mulli A Cyrys J Wichmann HE Peters A Runs of ventricular and supraventricular tachycardia triggered by air pollution in patients with coronary heart disease J Occup Environ Med 2006 48 11 1149 1158 10.1097/01.jom.0000245921.15916.03 17099451

[B3] de Hartog JJ Lanki T Timonen KL Hoek G Janssen NA Ibald-Mulli A Peters A Heinrich J Tarkiainen TH Van Grieken R van Wijnen JH Brunekreef B Pekkanen J Associations between PM2.5 and heart rate variability are modified by particle composition and beta-blocker use in patients with coronary heart disease Environ Health Perspect 2009 117 1 105 111 19165395 10.1289/ehp.11062 PMC2627852

[B4] Dockery DW Luttmann-Gibson H Rich DQ Link MS Mittleman MA Gold DR Koutrakis P Schwartz JD Verrier RL Association of air pollution with increased incidence of ventricular tachyarrhythmias recorded by implanted cardioverter defibrillators Environ Health Perspect 2005 113 6 670 674 10.1289/ehp.7767 15929887 PMC1257589

[B5] Dubowsky Adar S Gold DR Coull BA Schwartz J Stone PH Suh H Focused exposures to airborne traffic particles and heart rate variability in the elderly Epidemiology 2007 18 1 95 103 10.1097/01.ede.0000249409.81050.46 17149139

[B6] Ljungman PL Berglind N Holmgren C Gadler F Edvardsson N Pershagen G Rosenqvist M Sjogren B Bellander T Rapid effects of air pollution on ventricular arrhythmias Eur Heart J 2008 29 23 2894 2901 10.1093/eurheartj/ehn463 19004842

[B7] Peters A Liu E Verrier RL Schwartz J Gold DR Mittleman M Baliff J Oh JA Allen G Monahan K Dockery DW Air pollution and incidence of cardiac arrhythmia Epidemiology 2000 11 1 11 17 10.1097/00001648-200001000-00005 10615837

[B8] Peters A von Klot S Heier M Trentinaglia I Hormann A Wichmann HE Löwel H Exposure to traffic and the onset of myocardial infarction N Engl J Med 2004 351 17 1721 1730 10.1056/NEJMoa040203 15496621

[B9] Zareba W Nomura A Couderc JP Cardiovascular effects of air pollution: what to measure in ECG? Environ Health Perspect 2001 109 Suppl 4 533 538 10.2307/3454665 11544159 PMC1240577

[B10] Tsuji H Larson MG Venditti FJ Jr Manders ES Evans JC Feldman CL Levy D Impact of reduced heart rate variability on risk for cardiac events. The Framingham Heart Study Circulation 1996 94 11 2850 2855 8941112 10.1161/01.cir.94.11.2850

[B11] Nolan J Batin PD Andrews R Lindsay SJ Brooksby P Mullen M Baig W Flapan AD Cowley A Prescott RJ Neilson JM Fox KA Prospective study of heart rate variability and mortality in chronic heart failure: results of the United Kingdom heart failure evaluation and assessment of risk trial (UK-heart) Circulation 1998 98 15 1510 1516 9769304 10.1161/01.cir.98.15.1510

[B12] Bauer A Kantelhardt JW Bunde A Barthel P Schneider R Malik M Schmidt G Phase-rectified signal averaging detects quasi-periodicities in non-stationary data Physica A 2006 364 423 434 10.1016/j.physa.2005.08.080

[B13] Bauer A Kantelhardt JW Barthel P Schneider R Makikallio T Ulm K Hnatkova K Schomig A Huikuri H Bunde A Malik M Schmidt G Deceleration capacity of heart rate as a predictor of mortality after myocardial infarction: cohort study Lancet 2006 367 9523 1674 1681 10.1016/S0140-6736(06)68735-7 16714188

[B14] Chan CC Chuang KJ Shiao GM Lin LY Personal exposure to submicrometer particles and heart rate variability in human subjects Environ Health Perspect 2004 112 10 1063 1067 10.1289/ehp.6897 15238278 PMC1247378

[B15] Gold DR Litonjua A Schwartz J Lovett E Larson A Nearing B Allen G Verrier M Cherry R Verrier R Ambient pollution and heart rate variability Circulation 2000 101 11 1267 1273 10725286 10.1161/01.cir.101.11.1267

[B16] Park SK O'Neill MS Vokonas PS Sparrow D Schwartz J Effects of air pollution on heart rate variability: the VA normative aging study Environ Health Perspect 2005 113 3 304 309 10.1289/ehp.7447 15743719 PMC1253756

[B17] Wheeler A Zanobetti A Gold DR Schwartz J Stone P Suh HH The relationship between ambient air pollution and heart rate variability differs for individuals with heart and pulmonary disease Environ Health Perspect 2006 114 4 560 566 10.1289/ehp.8337 16581546 PMC1440781

[B18] de Hartog JJ Hoek G Peters A Timonen KL Ibald-Mulli A Brunekreef B Heinrich J Tiittanen P van Wijnen JH Kreyling W Kulmala M Pekkanen J Effects of fine and ultrafine particles on cardiorespiratory symptoms in elderly subjects with coronary heart disease: the ULTRA study Am J Epidemiol 2003 157 7 613 623 10.1093/aje/kwg021 12672681

[B19] Pekkanen J Peters A Hoek G Tiittanen P Brunekreef B de Hartog J Heinrich J Ibald-Mulli A Kreyling WG Lanki T Timonen KL Vanninen E Particulate air pollution and risk of ST-segment depression during repeated submaximal exercise tests among subjects with coronary heart disease: the Exposure and Risk Assessment for Fine and Ultrafine Particles in Ambient Air (ULTRA) study Circulation 2002 106 8 933 938 10.1161/01.CIR.0000027561.41736.3C 12186796

[B20] Wichmann HE Spix C Tuch T Wolke G Peters A Heinrich J Kreyling WG Heyder J Daily mortality and fine and ultrafine particles in Erfurt, Germany, part I: role of particle number and particle mass Res Rep Health Eff Inst 2000 98 5 86 11918089

[B21] Nemmar A Hoet PH Vanquickenborne B Dinsdale D Thomeer M Hoylaerts MF Vanbilloen H Mortelmans L Nemery B Passage of inhaled particles into the blood circulation in humans Circulation 2002 105 4 411 414 10.1161/hc0402.104118 11815420

[B22] Nemmar A Hoylaerts MF Hoet PH Nemery B Possible mechanisms of the cardiovascular effects of inhaled particles: systemic translocation and prothrombotic effects Toxicol Lett 2004 149 1-3 243 253 10.1016/j.toxlet.2003.12.061 15093270

[B23] Oberdorster G Sharp Z Atudorei V Elder A Gelein R Lunts A Kreyling W Cox C Extrapulmonary translocation of ultrafine carbon particles following whole-body inhalation exposure of rats J Toxicol Environ Health A 2002 65 20 1531 1543 10.1080/00984100290071658 12396867

[B24] Oberdorster G Sharp Z Atudorei V Elder A Gelein R Kreyling W Cox C Translocation of inhaled ultrafine particles to the brain Inhal Toxicol 2004 16 6-7 437 445 10.1080/08958370490439597 15204759

[B25] Zareba W Couderc JP Oberdorster G Chalupa D Cox C Huang LS Peters A Utell MJ Frampton MW ECG parameters and exposure to carbon ultrafine particles in young healthy subjects Inhal Toxicol 2009 21 3 223 233 10.1080/08958370802492407 18991063 PMC2867237

[B26] Malik M Heart rate variability: standards of measurement, physiological interpretation and clinical use. Task Force of the European Society of Cardiology and the North American Society of Pacing and Electrophysiology Circulation 1996 93 5 1043 1065 8598068

[B27] Peters A Breitner S Cyrys J Stölzel M Pitz M Wölke G Heinrich J Kreyling W Küchenhoff H Wichmann H-E Improved air quality and its influences on short-term health effects in Erfurt, Eastern Germany Res Rep Health Eff Inst 2009 137 5 90 19554968

[B28] Henneberger A Zareba W Ibald-Mulli A Ruckerl R Cyrys J Couderc JP Mykins B Woelke G Wichmann HE Peters A Repolarization changes induced by air pollution in ischemic heart disease patients Environ Health Perspect 2005 113 4 440 446 10.1289/ehp.7579 15811835 PMC1278484

[B29] Lombardi F Chaos theory, heart rate variability, and arrhythmic mortality Circulation 2000 101 1 8 10 10618296 10.1161/01.cir.101.1.8

[B30] Godleski JJ Verrier RL Koutrakis P Catalano P Coull B Reinisch U Lovett EG Lawrence J Murthy GG Wolfson JM Clarke RW Nearing BD Killingsworth C Mechanisms of morbidity and mortality from exposure to ambient air particles Res Rep Health Eff Inst 2000 91 5 88 10817681

[B31] Elder A Couderc JP Gelein R Eberly S Cox C Xia X Zareba W Hopke P Watts W Kittelson D Frampton M Utell M Oberdorster G Effects of on-road highway aerosol exposures on autonomic responses in aged, spontaneously hypertensive rats Inhal Toxicol 2007 19 1 1 12 10.1080/08958370600985735 17127638

[B32] Rhoden CR Wellenius GA Ghelfi E Lawrence J Gonzalez-Flecha B PM-induced cardiac oxidative stress and dysfunction are mediated by autonomic stimulation Biochim Biophys Acta 2005 1725 3 305 313 16005153 10.1016/j.bbagen.2005.05.025

[B33] Liao D Duan Y Whitsel EA Zheng ZJ Heiss G Chinchilli VM Lin HM Association of higher levels of ambient criteria pollutants with impaired cardiac autonomic control: a population-based study Am J Epidemiol 2004 159 8 768 777 10.1093/aje/kwh109 15051586

[B34] Peters A Perz S Doring A Stieber J Koenig W Wichmann HE Increases in heart rate during an air pollution episode Am J Epidemiol 1999 150 10 1094 1098 10568625 10.1093/oxfordjournals.aje.a009934

[B35] Pope CA III Verrier RL Lovett EG Larson AC Raizenne ME Kanner RE Schwartz J Villegas GM Gold DR Dockery DW Heart rate variability associated with particulate air pollution Am Heart J 1999 138 5 890 899 10.1016/S0002-8703(99)70014-1 10539820

[B36] Creason J Neas L Walsh D Williams R Sheldon L Liao D Shy C Particulate matter and heart rate variability among elderly retirees: the Baltimore 1998 PM study J Expo Anal Environ Epidemiol 2001 11 2 116 122 10.1038/sj.jea.7500154 11409004

[B37] Liao D Creason J Shy C Williams R Watts R Zweidinger R Daily variation of particulate air pollution and poor cardiac autonomic control in the elderly Environ Health Perspect 1999 107 7 521 525 10378998 10.1289/ehp.99107521 PMC1566669

[B38] Pope CA III Hansen ML Long RW Nielsen KR Eatough NL Wilson WE Eatough DJ Ambient particulate air pollution, heart rate variability, and blood markers of inflammation in a panel of elderly subjects Environ Health Perspect 2004 112 3 339 345 10.1289/ehp.6588 14998750 PMC1241864

[B39] Zanobetti A Gold DR Stone PH Suh HH Schwartz J Coull BA Speizer FE Reduction in heart rate variability with traffic and air pollution in patients with coronary artery disease Environ Health Perspect 2010 118 3 324 330 10.1289/ehp.0901003 20064780 PMC2854758

[B40] Devlin RB Ghio AJ Kehrl H Sanders G Cascio W Elderly humans exposed to concentrated air pollution particles have decreased heart rate variability Eur Respir J Suppl 2003 40 76s 80s 10.1183/09031936.03.00402403 12762579

[B41] Riediker M Cascio WE Griggs TR Herbst MC Bromberg PA Neas L Williams RW Devlin RB Particulate matter exposure in cars is associated with cardiovascular effects in healthy young men Am J Respir Crit Care Med 2004 169 8 934 940 10.1164/rccm.200310-1463OC 14962820

[B42] Samet JM Rappold A Graff D Cascio WE Berntsen JH Huang YC Herbst M Bassett M Montilla T Hazucha MJ Bromberg PA Devlin RB Concentrated ambient ultrafine particle exposure induces cardiac changes in young healthy volunteers Am J Respir Crit Care Med 2009 179 11 1034 1042 10.1164/rccm.200807-1043OC 19234105

[B43] Timonen KL Vanninen E de Hartog J Ibald-Mulli A Brunekreef B Gold DR Heinrich J Hoek G Lanki T Peters A Tarkiainen T Tiittanen P Kreyling W Pekkanen J Effects of ultrafine and fine particulate and gaseous air pollution on cardiac autonomic control in subjects with coronary artery disease: the ULTRA study J Expo Sci Environ Epidemiol 2006 16 4 332 341 10.1038/sj.jea.7500460 16205787

[B44] Chuang KJ Chan CC Chen NT Su TC Lin LY Effects of particle size fractions on reducing heart rate variability in cardiac and hypertensive patients Environ Health Perspect 2005 113 12 1693 1697 10.1289/ehp.8145 16330349 PMC1314907

[B45] Mauderly JL Chow JC Health effects of organic aerosols Inhal Toxicol 2008 20 3 257 288 10.1080/08958370701866008 18300047

[B46] Schwartz J Litonjua A Suh H Verrier M Zanobetti A Syring M Nearing B Verrier R Stone P MacCallum G Speizer FE Gold DR Traffic related pollution and heart rate variability in a panel of elderly subjects Thorax 2005 60 6 455 461 10.1136/thx.2004.024836 15923244 PMC1747419

[B47] Widdicombe J Lee LY Airway reflexes, autonomic function, and cardiovascular responses Environ Health Perspect 2001 109 Suppl 4 579 584 10.2307/3454673 11544167 PMC1240585

[B48] Utell MJ Frampton MW Zareba W Devlin RB Cascio WE Cardiovascular effects associated with air pollution: potential mechanisms and methods of testing Inhal Toxicol 2002 14 12 1231 1247 10.1080/08958370290084881 12454788

[B49] Schulz H Harder V Ibald-Mulli A Khandoga A Koenig W Krombach F Radykewicz R Stampfl A Thorand B Peters A Cardiovascular effects of fine and ultrafine particles J Aerosol Med 2005 18 1 1 22 10.1089/jam.2005.18.1 15741770

[B50] Graff DW Cascio WE Brackhan JA Devlin RB Metal particulate matter components affect gene expression and beat frequency of neonatal rat ventricular myocytes Environ Health Perspect 2004 112 7 792 798 15159208 10.1289/ehp.112-1241994 PMC1241994

[B51] Chuang KJ Chan CC Su TC Lee CT Tang CS The effect of urban air pollution on inflammation, oxidative stress, coagulation, and autonomic dysfunction in young adults Am J Respir Crit Care Med 2007 176 4 370 376 10.1164/rccm.200611-1627OC 17463411

[B52] Delfino RJ Staimer N Tjoa T Polidori A Arhami M Gillen DL Kleinman MT Vaziri ND Longhurst J Zaldivar F Sioutas C Circulating biomarkers of inflammation, antioxidant activity, and platelet activation are associated with primary combustion aerosols in subjects with coronary artery disease Environ Health Perspect 2008 116 7 898 906 10.1289/ehp.11189 18629312 PMC2453158

[B53] Dubowsky SD Suh H Schwartz J Coull BA Gold DR Diabetes, obesity, and hypertension may enhance associations between air pollution and markers of systemic inflammation Environ Health Perspect 2006 114 7 992 998 10.1289/ehp.8469 16835049 PMC1513328

[B54] Peters A Doring A Wichmann HE Koenig W Increased plasma viscosity during an air pollution episode: a link to mortality? Lancet 1997 349 9065 1582 1587 10.1016/S0140-6736(97)01211-7 9174559

[B55] Peters A Frohlich M Doring A Immervoll T Wichmann HE Hutchinson WL Pepys MB Koenig W Particulate air pollution is associated with an acute phase response in men; results from the MONICA-Augsburg Study Eur Heart J 2001 22 14 1198 1204 10.1053/euhj.2000.2483 11440492

[B56] Ruckerl R Ibald-Mulli A Koenig W Schneider A Woelke G Cyrys J Heinrich J Marder V Frampton M Wichmann HE Peters A Air pollution and markers of inflammation and coagulation in patients with coronary heart disease Am J Respir Crit Care Med 2006 173 4 432 441 10.1164/rccm.200507-1123OC 16293802

[B57] Donaldson K Tran L Jimenez LA Duffin R Newby DE Mills N MacNee W Stone V Combustion-derived nanoparticles: a review of their toxicology following inhalation exposure Part Fibre Toxicol 2005 2 10 10.1186/1743-8977-2-10 16242040 PMC1280930

[B58] Schwartz J Park SK O'Neill MS Vokonas PS Sparrow D Weiss S Kelsey K Glutathione-S-Transferase M1, Obesity, Statins, and Autonomic Effects of Particles: Gene-by-Drug-by-Environment Interaction Am J Respir Crit Care Med 2005 172 12 1529 1533 10.1164/rccm.200412-1698OC 16020798 PMC2718454

[B59] Xia T Kovochich M Nel A The role of reactive oxygen species and oxidative stress in mediating particulate matter injury Clin Occup Environ Med 2006 5 4 817 836 17110294 10.1016/j.coem.2006.07.005

[B60] Chuang KJ Coull BA Zanobetti A Suh H Schwartz J Stone PH Litonjua A Speizer FE Gold DR Particulate air pollution as a risk factor for ST-segment depression in patients with coronary artery disease Circulation 2008 118 13 1314 1320 10.1161/CIRCULATIONAHA.108.765669 18779445 PMC2751595

[B61] O'Neill MS Veves A Zanobetti A Sarnat JA Gold DR Economides PA Horton ES Schwartz J Diabetes enhances vulnerability to particulate air pollution-associated impairment in vascular reactivity and endothelial function Circulation 2005 111 22 2913 2920 10.1161/CIRCULATIONAHA.104.517110 15927967

[B62] Schneider A Neas L Herbst MC case m Williams RW Cascio W Hinderliter A Holguin F Buse JB Dungan K Styner M Peters A Devlin RB Endothelial dysfunction: associations with exposure to ambient fine particles in diabetic individuals Environ Health Perspect 2008 116 12 1666 1674 10.1289/ehp.11666 19079718 PMC2599761

[B63] Singer DH Ori Z Malik M, Camm AJ Changes in heart rate variability associated with sudden cardiac death Heart Rate Variability 1995 Armonk, Futura Publishing Company, Inc

[B64] de Bruyne MC Kors JA Hoes AW Klootwijk P Dekker JM Hofman A van Bemmel JH Grobbee DE Both decreased and increased heart rate variability on the standard 10-second electrocardiogram predict cardiac mortality in the elderly: the Rotterdam Study Am J Epidemiol 1999 150 12 1282 1288 10604770 10.1093/oxfordjournals.aje.a009959

[B65] Buccelletti E Gilardi E Scaini E Galiuto L Persiani R Biondi A Basile F Silveri NG Heart rate variability and myocardial infarction: systematic literature review and metanalysis Eur Rev Med Pharmacol Sci 2009 13 4 299 307 19694345

[B66] Dekker JM Crow RS Folsom AR Hannan PJ Liao D Swenne CA Schouten EG Low heart rate variability in a 2-minute rhythm strip predicts risk of coronary heart disease and mortality from several causes: the ARIC Study. Atherosclerosis Risk In Communities Circulation 2000 102 11 1239 1244 10982537 10.1161/01.cir.102.11.1239

[B67] Tsuji H Venditti FJ Jr Manders ES Evans JC Larson MG Feldman CL Levy D Reduced heart rate variability and mortality risk in an elderly cohort. The Framingham Heart Study Circulation 1994 90 2 878 883 8044959 10.1161/01.cir.90.2.878

[B68] Billman GE Schwartz PJ Stone HL Baroreceptor reflex control of heart rate: a predictor of sudden cardiac death Circulation 1982 66 4 874 880 7116603 10.1161/01.cir.66.4.874

[B69] Eckberg DL Drabinsky M Braunwald E Defective cardiac parasympathetic control in patients with heart disease N Engl J Med 1971 285 16 877 883 10.1056/NEJM197110142851602 4398792

[B70] Schwartz PJ La Rovere MT Vanoli E Autonomic nervous system and sudden cardiac death. Experimental basis and clinical observations for post-myocardial infarction risk stratification Circulation 1992 85 Suppl 1 I77 I91 1728509

[B71] Malliani A Lombardi F Pagani M Cerutti S Power spectral analysis of cardiovascular variability in patients at risk for sudden cardiac death J Cardiovasc Electrophysiol 1994 5 3 274 286 10.1111/j.1540-8167.1994.tb01164.x 8193742

[B72] Pozzati A Pancaldi LG Di Pasquale G Pinelli G Bugiardini R Transient sympathovagal imbalance triggers "ischemic" sudden death in patients undergoing electrocardiographic Holter monitoring J Am Coll Cardiol 1996 27 4 847 852 10.1016/0735-1097(96)00033-2 8613613

[B73] Zeger SL Thomas D Dominici F Samet JM Schwartz J Dockery D Cohen A Exposure measurement error in time-series studies of air pollution: concepts and consequences Environ Health Perspect 2000 108 5 419 426 10.2307/3454382 10811568 PMC1638034

