# Supplementary material for: Changes in deceleration capacity of heart rate and heart rate variability induced by ambient air pollution in individuals with coronary artery disease
Source: Part Fibre Toxicol. 2010 Oct 7;7:29. doi: 10.1186/1743-8977-7-29 (PMC2958976; doi:10.1186/1743-8977-7-29)
Supplement: Additional file 1 — Selected confounder models for the ECG-parameters in the short-term and the long-term recordings. [file 1743-8977-7-29-S1.PDF]

Online Supplemental Material: Additional File 1

Selected confounder models for the ECG parameters in the short-term and the long-term recordings:

5-minute recordings

HF

	Lag	Functional form
**Time trend**		linear
**Air temperature**	1	linear
**Relative humidity**	2	linear
**Barometric pressure**	-	-
**Day of the week**	-	-

LF

	Lag	Functional form
**Time trend**		linear
**Air temperature**	3	linear
**Relative humidity**	0	linear
**Barometric pressure**	0	linear
**Day of the week**	-	-

lnRMSSD

	Lag	Functional form
**Time trend**		linear
**Air temperature**	1	linear
**Relative humidity**	1	linear
**Barometric pressure**	-	-
**Day of the week**	-	-

HR

	Lag	Functional form
**Time trend**		kubisch
**Air temperature**	0	linear
**Relative humidity**	1	linear
**Barometric pressure**	-	-
**Day of the week**	-	-

<u>24-hour recordings</u>

lnRMSSD

	Lag	Functional form
**Time trend**		linear
**Air temperature**	1	linear
**Relative humidity**	2	linear
**Barometric pressure**	2	linear
**Day of the week**	-	-

SDNN

	Lag	Functional form
**Time trend**		linear
**Air temperature**	0	linear
**Relative humidity**	1	linear
**Barometric pressure**	-	-
**Day of the week**	-	-

HR

	Lag	Functional form
**Time trend**		linear
**Air temperature**	conc.	linear
**Relative humidity**	1	linear
**Barometric pressure**	-	-
**Day of the week**	-	-

pNN50

	Lag	Functional form
**Time trend**		linear
**Air temperature**	1	linear
**Relative humidity**	conc.	linear
**Barometric pressure**	-	-
**Day of the week**	-	-

DC

	Lag	Functional form
**Time trend**		linear
**Air temperature**	2	kubisch
**Relative humidity**	2	linear
**Barometric pressure**	0	linear
**Day of the week**	-	-
