# Supplementary material for: Changes in deceleration capacity of heart rate and heart rate variability induced by ambient air pollution in individuals with coronary artery disease
Source: Part Fibre Toxicol. 2010 Oct 7;7:29. doi: 10.1186/1743-8977-7-29 (PMC2958976; doi:10.1186/1743-8977-7-29)
Supplement: Additional file 2 — Effect estimates with 95%-confidence intervals based on interquartile range increases in air pollutants for the interaction analyses with times spent in traffic (using a car, bus, tram or taxi). [file 1743-8977-7-29-S2.PDF]

Online Supplemental Material: Additional File 2

Effect estimates with 95%-confidence intervals based on interquartile range increases in air pollutants for the interaction analyses with times spent in traffic (using a car, bus, tram or taxi):

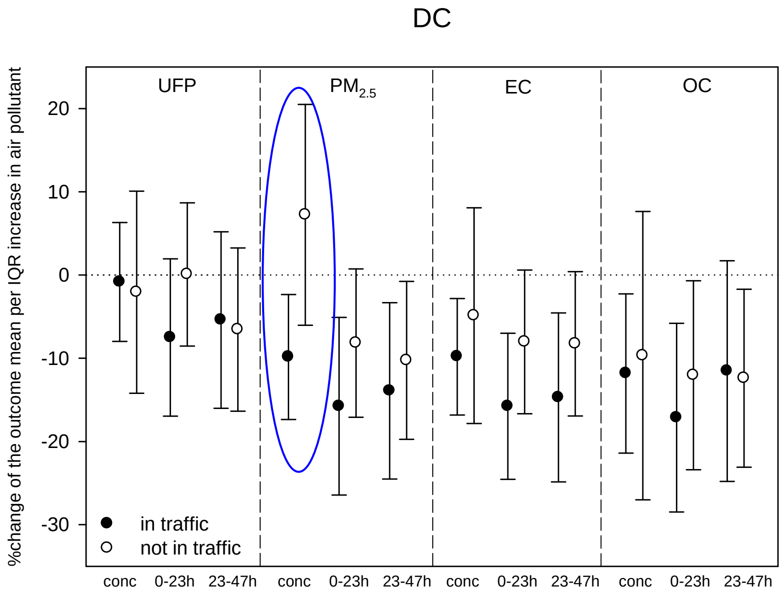

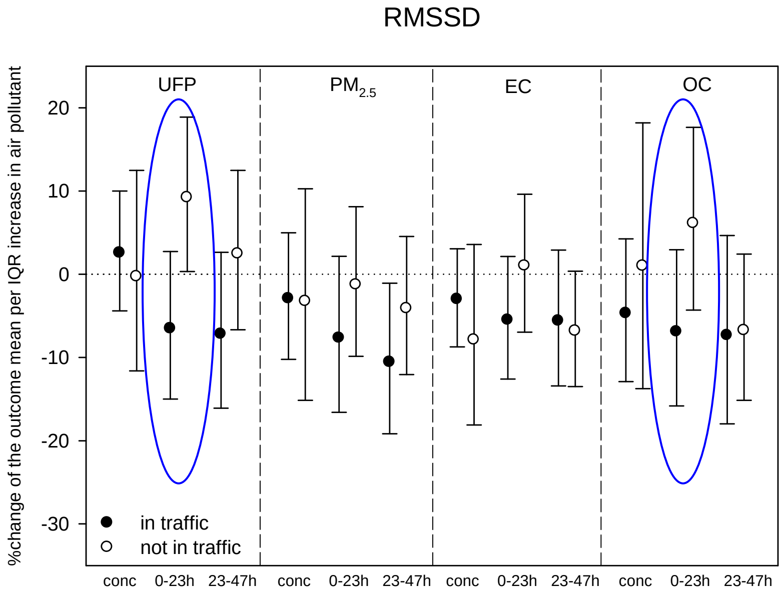

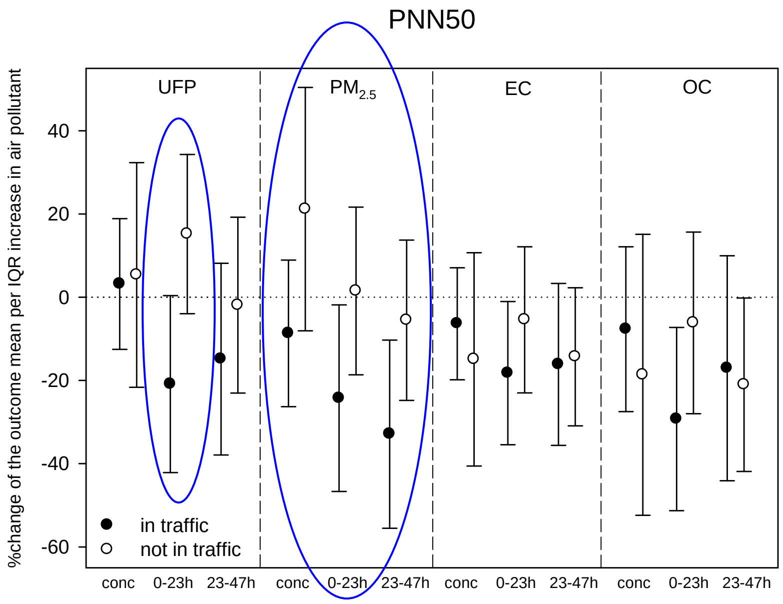

Significant ($p<0.05$) as well as borderline significant ($p<0.10$) interactions are marked with a blue circle.

For the 254 24-hour ECGs the following numbers of observations were observed for times spent in traffic:

**Times spent in traffic**	During 24-hour ECG (conc.)	24 hours before ECG (0-23h)	48 hours before ECG (23-47h)
Less than one hour	68	120	131
More than one hour	169	119	109
Information missing	17	15	14

In traffic: at least one hour in traffic
Not in traffic: less than one hour in traffic
IQR: interquartile range
UFP: number concentration of particles between 0.01 and 0.1µm diameter
$PM_{2.5}$: mass concentration of particles <2.5 µm diameter
EC and OC: elemental and organic carbon
DC: deceleration capacity
RMSSD: square root of the mean square of successive differences of normal-to-normal (NN) intervals
pNN50: percentage of adjacent NN intervals which differ more than 50ms
conc.: concurrent to the ECG-measurements
0-23h: 0-23 hours before the ECG-measurements
23-47h: 23-47 hours before the ECG-measurements
